# Suspension of face-to-face teaching and ad hoc transition to digital learning under Covid-19 conditions – a qualitative study among dental students and lecturers

**DOI:** 10.1186/s12909-022-03335-5

**Published:** 2022-04-08

**Authors:** Katrin Hertrampf, Hans-Jürgen Wenz, Hanna Kaduszkiewicz, Katja Goetz

**Affiliations:** 1 Department of Oral and Maxillofacial Surgery, University Hospital of Schleswig-Holstein, Campus Kiel Arnold-Heller Str. 3, Haus 26, 24105 Kiel, Germany; 2 Department of Prosthodontics, Propaedeutics and Dental Materials, University Hospital of Schleswig-Holstein, Campus Kiel, Kiel, Germany; 3 Institute of General Practice, University of Kiel, Kiel, Germany; 4 Institute of Family Medicine, University Hospital of Schleswig-Holstein, CampusLübeck, Lübeck, Germany

**Keywords:** Covid-19, Dental education, Digitalisation, Students' experiences, Teachers, Experiences

## Abstract

**Background:**

In mid-March 2020, the coronavirus pandemic led to a national lockdown in Germany. Face-to-face teaching was cancelled in universities for the 2020 summer semester. Teaching moved online with no prior IT testing and lecturer training. The study analyses experiences of the suspension of face-to-face teaching and the move to digitalised learning for students and lecturers of dentistry at Kiel.

**Methods:**

In summer 2020, qualitative guided interviews were conducted with students (4th, 6th, 8th, and 10th semesters), and lecturers. Deductive and inductive qualitative content analysis of the results was carried out.

**Results:**

Thirty-nine students (69% female) and 19 lecturers (32% female) were interviewed. Reactions to the changes in teaching were observed. Feelings ranged from an essentially positive attitude, through insecurity and uncertainty to a failure to fully appreciate the situation. The loss of social contact was lamented. Digitalisation was associated with technological challenges and additional work. However, it also fostered learning independent of time and place, and encouraged autonomy. Negative aspects of digitalisation included a lack of feedback and loss of interaction.

**Conclusion:**

The introduction of ad hoc digitalisation challenged both students and lecturers alike. Dealing with lockdown and the changes in teaching and studying required significant flexibility.

**Supplementary Information:**

The online version contains supplementary material available at 10.1186/s12909-022-03335-5.

## Introduction

In December 2019, an outbreak of a new coronavirus was reported in Wuhan, China. Following China, several European countries reported increasing numbers of infections, with the virus spreading worldwide and declared a Public Health Emergency of International Concern by the WHO at the end of January 2020 [1, 2]. By the end of February 2020, over 80,000 infections and approximately 2,700 deaths had been reported in 34 countries [3]. Since then, 265,108,084 positive cases and 5,246.724 deaths have been reported worldwide [4]. Due to the coronavirus pandemic, Germany, in common with many other countries, instituted a national shutdown from mid March 2020 with gradual relaxations from mid May. In order to reduce infections, social distancing regulations were introduced in public and private life [5]. For the university sector, this meant suspending face-to-face teaching for the 2020 summer semester and switching to digital teaching formats to protect students, teachers, administrative staff, and patients [6–8]. In Germany, these changes had to be carried out on a broad scale over a very short period without prior implementation and testing of the required IT infrastructure. While prior training and induction for lecturers in digital teaching formats would have been desirable, it was not possible. Since dental education is very practically oriented, incorporating patient treatment, and is almost completely conducted live, there was little to no experience with digital teaching methods.

Thus, due to the very narrow window from mid March to the beginning of April (the start of the summer semester), a step-by-step approach (where training follows implementation) was not feasible and processes ran in parallel or even in reverse order.

The combination of an ad hoc transition to digitalised teaching and the uncertainty of a pandemic confronted all those involved, both teachers and learners alike, with hitherto unfamiliar challenges, as "forced distance learning" took the place of close contact and communication, and solution strategies were implemented internationally in accordance with respective country-specific circumstances [9]. Challenges were particularly acutely felt in the case of dental education since the usual early ongoing patient contact does not appear to be able to be digitally replicated. The way in which teachers and students have experienced and assess this digital ad hoc implementation has not yet been sufficiently investigated [10, 11].

The aim of the study was thus to explore the impact of the coronavirus pandemic on the teaching of dentistry at Kiel in terms of assessments, experiences, obstacles, and barriers from the perspective of dental students and their lecturers. The focus of the study was on the suspension of face-to-face teaching and the ad hoc digitalisation of teaching.

## Materials and methods

### Study design

The present study was designed as a qualitative study with a grounded theory approach to explore the experiences of students and teaching staff regarding the suspension of face-to-face teaching and ad hoc digitalisation. The COREQ checklist for comprehensive reporting of qualitative studies was used [12] (Additional file 1).

### The dental programm

In Germany, the study of dentistry is a five-year program, followed by approximately six months of state examinations, which include both oral examinations and practical examinations on patients. After the 2nd and 5th semesters, oral state examinations are also given in the basic medical subjects, as well as a practical examination on the phantom. In the clinical study (6th-10th semester), a large part of the curriculum consists of treating patients, with a clear emphasis on preventive and restorative dentistry, periodontics, and prosthodontics.

### State of teaching

Lectures were published permanently as streams on the university's previously established OLAT teaching platform. Most departments also offered uninterrupted video conferences for students. Students were invited to send questions in advance of events. Two lectures were held as live streams. Seminars were conducted as video conferences. As was the case before the pandemic, the majority of the theoretical assessment was conducted via written examination. In a few cases, no exam was written. Theoretical performance overall was comparable to that before the pandemic.

Later in the semester and extending the courses into the semester break, it was possible to start face-to-face teaching again in the preclinical simulation courses and in the clinical patient courses under very strict hygienic and organizational conditions. Of all practical university teaching, only the dental courses were granted an exemption from the university. The implications of the COVID-19 pandemic on the practical teaching will be the subject of another paper.

### Recruitment

Students were recruited from courses held in the last third of the summer semester via video conference. Lecturers from the various departments were given personal presentations on the project. Participation was voluntary for all. Appointments for the interviews were then made in person or by email. Data collection took place between June and August 2020. All interviews were conducted by two female members of the working group (KH, KG) either in person or by telephone. Both were experienced in performing qualitative research. As described in the literature, no difference in data quality was observed between face-to-face and telephone interviewing, and both may be recommended for use in the same qualitative study [13].

All interviews and minuting adhered to the same predefined quality criteria, for example with documentation of time and of any issues or interruptions encountered during the interview. Sociodemographic data was requested from participants before the start of each interview.

### Participants

Qualitative interviews were conducted with a purposive sample of students and teaching staff. Students from semesters four, six, eight and ten at the dental school in Kiel, Germany were included in the study along with associated teaching staff.

Dental simulation courses took place in the 4th and 6th semesters and clinical treatment courses in the 7th–10th semesters. The 8th and 10th semesters were selected as examples for the treatment courses. The target sample consisted of 10 students from each of the four specialist semesters along with the lecturers from the four departments and the departmental directors (*n* = 19 in total), taking into account contrast or saturation [14] (Patton 1990).

For the students, the following inclusion criteria were applied: membership of the respective subject semesters, being of age, and sufficient knowledge of the German language. For the lecturers, inclusion criteria were: responsibility for teaching content and its implementation in one of the Dental Clinic’s four departments, being of age, and sufficient knowledge of the German language.

### Data collection

A semi-structured interview guide was developed by an interdisciplinary team of a sociologist, health services researcher, physician, and dental practitioners. Following a literature review and discussion within the study team, the interview guide focussed on two main topics:

-Experiences of the suspension of face-to-face teaching.
-Experiences of ad hoc digitalisation.

The interview guides for students and teaching staff were identical (please see Additional file 2). The guides were tested with a student and lecturer for comprehensibility and the sequencing of individual questions.

### Data analysis

Interview duration varied and was about 31 min on average for the dental student group (min. = 22 min, max. = 50 min) and about 31 min for the teaching staff (min. = 15 min, max. = 41 min). All interviews were digitally audio recorded and transcribed in full verbatim. Transcripts were not submitted to participants for comments or correction. The texts were anonymised during transcription before undergoing qualitative content analysis [15].The ATLAS.ti 8.4 (Scientific Software Development GmbH, 2020) software was used to assist with data analysis. The research team used a deductive-inductive approach to generating thematic categories. Firstly, a provisional category system was developed deductively based on the interview guidelines. The provisional category system was then adjusted during analysis according to the content of the transcripts. Any new categories which emerged were then added following an inductive approach. Transcripts were coded independently into main and sub-categories by two researchers (KH [dental practitioner background] and KG [health services research background]), following intensive discussions which continued until consensus was achieved. Participant quotations were translated from German into English for publication purposes.

### Ethical approval

The project was approved by the Ethics Committee of the University of Kiel, Germany (D509/20) and was conducted in accordance with the Declaration of Helsinki. Informed consent was obtained via a signed consent form which included permission to publish anonymised quotes.

## Results

### Sample characteristics

Fifty-eight interviews were performed in total: 39 with dental students and 19 with teaching staff. Participant characteristics are shown in Table 1. The following sections describe the two main topics “experiences of suspension of face-to-face teaching” and “experiences of ad hoc digitalisation”. Quotations are used to illustrate relevant aspects reported by the participants (students [S] and teaching staff [TS]).

**Table 1:** Distribution of the participants

Variable	Students (*N* = 39)	Lecturer (*N* = 19)
**Women**	27	6
**Men**	12	13
**Age (Mean)**	25.2	44.0
**(Age span)**	(20–31)	(31–65)
**Apprenticeship**	13	-
**Further completed study**	5	-
**Additional qualification**	5	-
**Director of the clinic**	-	4
**Course instructor**	-	7
**Course assistent**	-	8
**Use of hardware**^a^**:**		
** Laptop**	30	18
** Tablet**	10	0
** Mobile phone**	3	0
** Stationary PC**	2	2
** Access to camera (yes)**	39	17
** Access to microphone (yes)**	38	17
** Permanent availability**	38	17
** Adequate internet connection (yes)**	38	19
** Course in homeoffice**		2

^a^Multiple answers possible

### Main topic: Experiences of suspension of face-to-face teaching

**Fig. 1:**
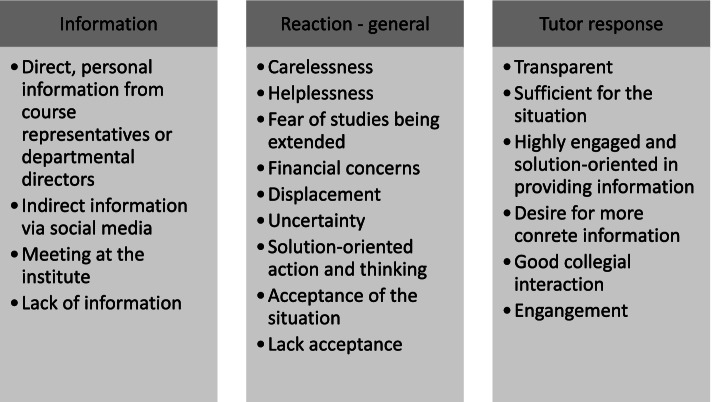
Experiences of the suspension of face-to-face teaching: information and response – main and sub-categories

**Fig. 2:**
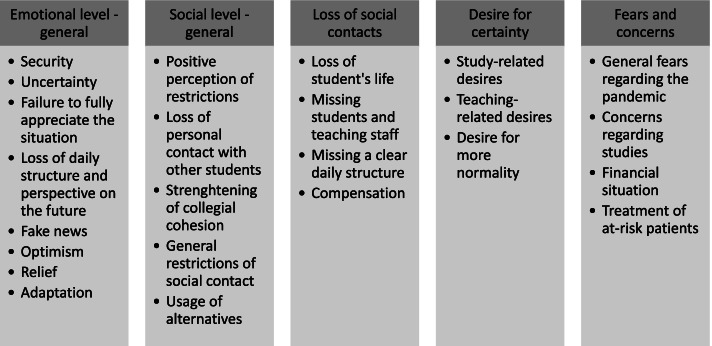
Experiences of the suspension of face-to-face teaching: emotional and social aspects – main and sub-categories

This topic describes how students and teaching staff were informed that face-to-face teaching was to be suspended, how they responded to this information, and how they were impacted at the emotional and social level. Due to the wealth of main and sub-categories, we decided to split the main categories between different figures. For this topic, eight main categories were created and divided into different sub-categories as shown in figs. 1 and 2.

**Fig. 3:**
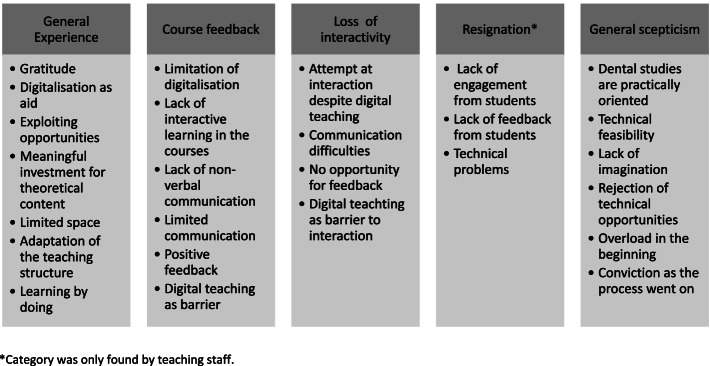
Experiences of ad hoc digitalisation: general aspects – main and sub-categories

**Fig. 4:**
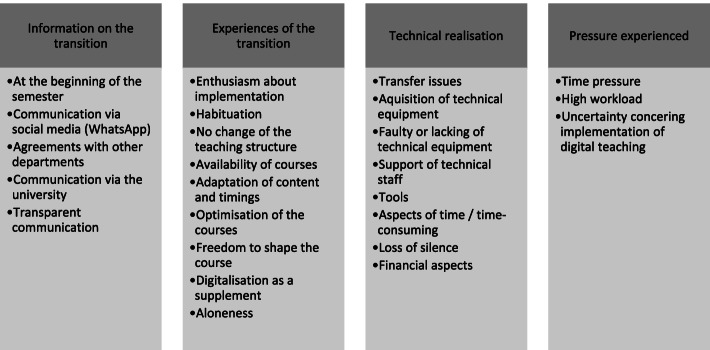
Experiences of ad hoc digitalisation: aspects of the transition to digital teaching – main and sub-categories

**Fig. 5:**
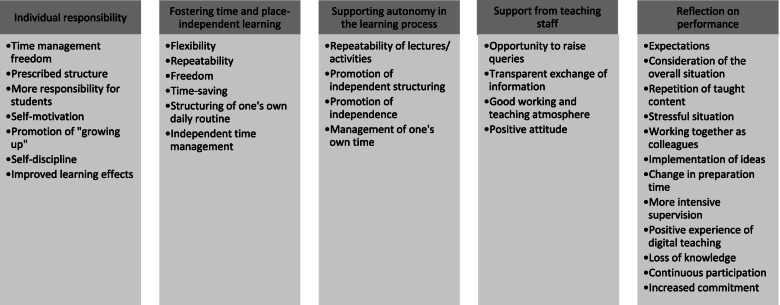
Experiences of ad hoc digitalisation: aspects of the specific teaching and learning situation – main and sub-categories

#### Information and response

The information (main category) provided to students and teaching staff about the suspension of face-to-face teaching was received through different communication channels including social media, tutors, the departmental director, and course representatives. The response (main category) to the suspension of face-to-face teaching included different sub-categories. Some students and teaching staff expressed a feeling of indifference: “In that sense, it wasn't so bad. Well, because you knew that at some point things had to go on again somehow” (S34). Some experienced helplessness: “Yes, I saw it as a disaster because you can't train dentists like that” (TS18). Students and teaching staff had concerns, including financial ones, about courses being extended: “So I thought, OK, it would be a bit dumb if I had to study for another half a year now.” (S20). However, some participants were surprised, and their reactions may be identified as a kind of displacement: “It was a surprise to me at first, because we work very practically” (TS06).

The main category “tutor response” describes how teaching staff reacted to the suspension of face-to-face teaching. Their response was mainly described as transparent and sufficient for the situation: “Very responsible … yeah, and very organised … yeah, so you felt well supported at that point.” (S10). Moreover, both students and teaching staff stated that tutors were highly engaged and solution-oriented in the way they provided information about the situation. “I think we actually solved it very well and very quickly, because the decision was very quickly taken to say, OK, we’ll first make sure that the theoretical teaching is done online” (TS04). Only some of the students felt the need for more concrete information: “But there was also this big question mark. A big question mark for everyone because you didn't know how things were going to continue” (S01).

#### Emotional and social aspects

Additional main and sub-categories for the topic “experiences of the suspension of face-to-face teaching” are presented in Fig. 2, which focuses on the different emotional and social aspects related to the suspension of face-to-face teaching.

For the main category “emotional level” a broad spectrum of feelings was observed, ranging from a feeling of security to uncertainty and a failure to fully appreciate the situation. By way of example, one student stated: “But I’ve actually always felt well taken care of” (S11). Feelings of uncertainty due to the suspension were expressed by both groups, students and teaching staff: “There was uncertainty too, of course – a lot at first, because you didn't know what was going to happen next” (TS06). Participating students, in particular, stated that they lost their daily routine and perspective on the future due to the lack of face-to-face teaching courses at the university. At the beginning of the semester, some students thought the information about the suspension was fake news: “[…]no one knew if it was fake or not” (S02).

The main categories “social level” and “loss of social contacts” described the social restrictions which were perceived due to the suspension of face-to-face teaching. These were highlighted more by students than by teaching staff. The “social level” included sub-categories such as a positive perception of restrictions from the perspective of students who saw the suspension as an extension of the holidays. Students often reported that they missed their student life and had lost personal contacts: “You may have WhatsApp or Zoom, but that's no substitute for meeting in person” (S21). Teaching staff reported that collegial cohesion has been strengthened due to this specific situation. They supported each other straightforwardly: “If someone said, ‘I need help as a course leader setting up my planning’, or ‘We have to make the videos here – who’s going to do which job?’ everyone was really helpful” (TS16).

“Loss of social contacts” explicitly emphasised the restrictions on social contact due to the suspension. Students missed the face-to-face contact with other fellow students and with the teaching staff. These aspects went hand in hand with the loss of student life. One student hit upon a clear description of the current situation:

“So it's very strange, because you just don't have that … that feeling that you’re studying any more” (S10).

The desire for certainty, one of the other main categories, included study-related and teaching-related desires. These desires related to clearer communication of how dental studies were going to be organised from the student’s perspective. The teaching staff primarily found teaching online to be a new challenge confronting them and would have like a more informed approach to the online platform. In general, students reported the desire for more normality:

“Someone to tell me that at this point, it'll all be over. That is, for there to be a point in time when you know that things will go back to normal” (S10).

The main category “fears and concerns” was given to general fears relating to the pandemic, and concerns regarding course and financial situations and the treatment of at-risk patients.

Students often mentioned their general anxiety, saying: “[…] of course, this kind of thing pulls the rug out from under you somewhat” (S29).

Students and teaching staff expressed their concerns regarding the course since the dental studies programme is a very practical course. One member of teaching staff reported: “How is the semester supposed to carry on? How is the course supposed to continue for the students if we now have to suspend a semester, so to speak?” (TS13). Students mainly had concerns regarding their financial situation due to dependence on governmental and/or parental financial support or the loss of their student job: “Yes, at first, because then you’ll be studying for half a year longer and you won't get any more financial support – I'm on BAföG [government support], not dependent on parents” (S06).

### Main topic: Experiences of ad hoc digitalisation

For this topic, 14 main categories emerged and were divided into different sub-categories, as shown in figs. 3, 4 and 5.

#### General aspects

Ad hoc digitalisation of teaching content was greatly appreciated by the students. The lecturers were also aware of this appreciation: “I felt a great deal of gratitude from the students, who were themselves unsure as to how things were going to proceed with my course” (TS11).

Digitalisation was also seen as a useful aid in the delivery of the semester: “It was just like sitting there in the semester, the semester before. With a few exceptions, it was really good” (S23); “Yes […] it is good. It works […] It's just not like a face-to-face activity, but with all the difficulties, I think it's a very good solution” (TS01).

However, students' and lecturers' statements also revealed the negative aspects of digitalisation. These ranged from a lack of feedback on courses, to a loss of interaction, itself leading to resignation on the part of the lecturers: “And also because I’m getting zero feedback from my courses at the moment” (TS02). Students and lecturers additionally perceived digital teaching as an obstacle. Students were more inhibited when it came to asking questions: “But there was a bit of a lack of direct interaction, and it’s much harder to ask questions” (S02). The lecturers, on the other hand, mainly commented on the lack of direct contact with the students: “Well, I found it very difficult to assess whether the other person was listening with interest […] Direct contact like this was really lacking because the students couldn’t speak either” (TS06).

#### Aspects of the transition to digital teaching

Notification of the fact that teaching would be digital from the summer semester 2020 was provided via various channels, either via the email distribution lists in the individual departments or centrally via email from university management. As a rule, both students and lecturers found the communication on this to be transparent. The response to the transition to digital teaching ranged from delight at how the teaching was being implemented to a feeling of being left to one's own devices.

In particular, student comments showed that the permanent availability of lecture recordings was a useful aid to the learning process or in preparing for exams: “It was all positive in the end actually, because – especially before written or oral exams – you could really go over lectures again” (S21). In addition, students said that this change had given them a little more freedom to organise things:

“Overall, I would see it as a good thing. I really felt you were a bit freer in your decisions and in the priorities you set in your learning” (S35). The lecturers' comments, on the other hand, showed that the freedom to make changes was rather difficult to make the most of due to everyday responsibilities:

“Digitalisation often suffered from the fact that, due to everyday busyness, you didn’t have the freedom to engage with it unless it was absolutely necessary” (TS13).

The ad hoc digitalisation of teaching was accompanied by challenges that mainly concerned its technological implementation. In addition to transmission problems due to maximum email attachment size limitations, or a lack of equipment such as cameras or external microphones, it was the lecturers above all who found the on-site support from qualified personnel helpful: “We actually have an IT technician right in our department. He provided us with a room where a laptop, microphone, and camera – everything! – was set up” (TS06). The change also led to an additional burden for the lecturers, both in terms of perceived time pressure and additional workload: “Except that it was so exhausting, and we haven’t had any training for this and some people here have been working, working, working overtime and they’ve had more and more put on them” (TS16). In addition, students expressed uncertainty about the implementation of digital teaching: “Only, what was overwhelming was that you didn't really know which subject had already been digitalised and what you needed to look at” (S27).

#### Teaching and learning specific aspects

The students also expressed that digitalisation was associated with a certain degree of personal responsibility and, at the same time, a promotion of time- and location-independent learning. Autonomy within the learning process was also fostered. The students experienced this personal responsibility as both a positive and a negative. In addition to being able to freely organise their time due to the digital availability of teaching materials, students also identified personal responsibility and better learning being fostered: “It was a positive that you were able to do theoretical work during your most productive periods, but a negative that if you don't have any limits on when something has to be finished, sometimes it’s difficult to really get down to it when it's a lot of work" (S26). The lecturers' comments showed above all that students’ responsibility for their own learning had increased: “There was a lot of personal responsibility for the students” (TS06). Due to digitalisation, there was greater promotion of learning independent of time and place, which was perceived positively by both students and lecturers. In addition to flexibility, mention was made of the opportunity to structure your daily routine independently, the increased freedom, and the possibility of repeating lecture content:

“In principle, I think digitalised seminars and lectures are a real bonus, because you’re also giving the students more flexibility” (TS03); “Well, I have to say that this semester has helped me a lot with digitalisation, and I’ve also realised that it’s beneficial to me, because I’ve been able to organise my daily schedule independently” (S36).

The support afforded by teaching staff included providing the ability to ask questions, the creation of a good working and teaching atmosphere, and transparent sharing of information. In some subjects, students were also given the opportunity to ask questions via Zoom: “And we also have a meeting with Prof. X once a week where we can discuss questions and send them to him by email” (S10).

Students and teachers alike recognised the essentially positive mood. This is shown by the following example statement from a student: "So I thought people were very accommodating, actually. I actually found it all pretty positive” (S23). Alongside demonstrating their expectations of themselves and their positive experiences of digital teaching, the students' desire to go back over taught content showed their reflection on their own performance: “But this semester, I think I still have to catch up on some things during the semester break” (S30). Lecturers felt that the preparation time for lectures had changed, that supervision of students by lecturers had become more intensive and that their own workload had increased as a result: “We’re already making so many exceptions with digitalisation and we’re also making a big effort to look after the students” (TS07).

## Discussion

The coronavirus pandemic posed great challenges for university teaching, especially dental teaching. The abrupt change from analogue to fully digital teaching confronted students and lecturers with various experiences, perceptions, and hurdles. The results of the qualitative study that we conducted highlight interesting themes regarding how students and lecturers experienced the suspension of face-to-face teaching and the ad hoc changeover.

With regard to the overarching responses to the suspension of face-to-face teaching and the transition to digital teaching, the picture among both students and lecturers was a rather heterogeneous mixture of feelings characterised by uncertainty, helplessness, indifference and even suppression of the thought of the situation, especially with regard to the very practical side of dental training. It is precisely this uncertainty, combined with questions as to ongoing implications for dental studies and of whether they will be prolonged as a result, that has also been described in other studies [7, 16, 17].

The majority of students viewed the practical implementation of digital learning by the teaching staff in Kiel as transparent, constructive, solution-oriented, and supportive. Similar positive feedback was also reported by Schlenz et al. [11]. Nevertheless, both this study and another [7] also noted the loss of daily structure due to the lack of student attendance on-site.

Students additionally complained about the lack of social contact, both as it concerns the resulting lack of student life and of face-to-face contact with their teachers and interaction with their fellow students in the dental clinic. These aspects, which ran like a thread through the interviews and were also perceived as a major loss by the teachers, were also observed in other studies [7, 11, 18].

Another important aspect for some of the participating students in our study was financial concern caused by the loss of a student job or the fear of losing state funding. In view of this, it is understandable that a desire for normality was expressed during the interviews.

The students expressed significant gratitude for the relative speed and innovation of the transition to digital teaching content, something which was also noted by the lecturers. Similar expressions of gratitude were also explicitly described in the qualitative study by Pietro et al. (2021). Furthermore, digitalisation was seen as a useful aid to allowing the semester to happen. The switch to digital teaching was also viewed in other studies [7, 11] as an opportunity for the development of digital teaching concepts.

However, students and lecturers also reported negative aspects to the way digitalisation had been implemented. One aspect that was of great concern to the lecturers was the lack of feedback provided on courses, despite requests. Elsewhere, attempts were made to exchange information about platforms implemented or how existing platforms had been developed, something which was described as positive [7, 18].

Another aspect criticised and lamented by both parties was the lack of interaction.

This led to resignation on the part of the lecturers. This loss of interaction has also been described in other studies [7, 9, 17, 19, 20]. Students clearly reported inhibitions about asking questions in this regard. These experiences were also described by Hattar et al. As a consequence, the authors emphasise the importance of continuous interaction between teachers and students [18]. Other working groups specifically recommended regular video conferencing for group discussion [17, 19]. However, it has also been pointed out that as good a medium as video conferencing is, it is no substitute for face-to-face interaction [7].

One very important positive aspect for the majority of students, also confirmed by other studies, was the permanent availability of lecture recordings as a useful support in the learning process or in preparing for exams. This leads to flexibility and greater freedom for students to organise their own learning [7, 9, 17, 20, 21]. However, there was disagreement over whether students have more time for learning as a result or whether it provides an additional burden [7, 19, 20].

With regard to possible additional burdens, the teachers, on the other hand, found that it was somewhat difficult to free up space for the changeover due to the daily workload created by the pandemic. This significant additional workload was also expressed by the Pietro et al. working group [7].

The ad hoc digitalisation of teaching was associated with various challenges, mainly concerning its technological implementation. However, the potential lack of influence over this crucial aspect has been criticised in other studies [9, 17, 19]. This uncertainty and dissatisfaction may be due to insufficient training and IT structures. Institutions that had these elements in place prior to implementation described higher satisfaction among teachers [9, 20].

With regard to the teaching and learning-specific aspects, the students in our study reported digitalisation being associated with a certain degree of personal responsibility which definitely encourages time- and location-independent learning. It may have been these benefits of permanent location-independent availability and the associated personal responsibility, as well as the improved learning effects, which led to the very rapid acceptance of the abrupt switch to digital teaching [7, 20, 21]. But the students did not always have a predominantly positive view of this either [19].

### Limitations and strengths

The results of this qualitative study were based on self-reporting, namely on the subjective comments of the participating students and lecturers. It is therefore not possible to make any assessments about the truthfulness of the information.

Since participation was voluntary, it must also be assumed that the study attracted interested students who were more open to the topics discussed. This “positive selection bias” may be reflected in the results and thus needs to be taken into account during interpretation.

Furthermore, it should be noted that the design of this qualitative study does not permit any generalisation of results. The desired participant numbers and thus saturation were, however, achieved.

The study’s quality was consistently ensured through adherence to pre-determined quality standards. All interviews were conducted by the same two individuals, using the same interview guidelines for all students under the same conditions. In addition to this, the proceedings from each interview were documented according to a previously agreed protocol.

Another strength of this study is that students and lecturers were surveyed regarding the same aspects during the interview. This meant that shared as well as differing perspectives could easily be identified.

## Conclusion

This qualitative study provides interesting insights into the experiences and perceptions of students and lecturers in the context of the suspension of face-to-face teaching and ad hoc digitalisation. The option of online teaching was very much welcomed, especially by the students, as it did not lead to studies being prolonged as feared. Online teaching, however, is no replacement for face-to-face interaction. It also became clear that both sides, students and lecturers, saw the lack of interaction as a loss. Maintaining an ongoing connection with students was a challenge, especially for the lecturers. As our results also show, investment should be made in suitable IT infrastructure to allow adequate preparations to be made in future.

### Practical implications

An adequate IT-infrastructure including well-functioned hardware and software would be necessary for teaching staff and students for performing and participation on digital teaching. Sufficient time and good preparation to prepare learning content digitally should be of future interest to those responsible for teaching. The expectations of the content of teaching, taking digital teaching formats into account, will change university didactics in the future.

## Supplementary Information

**Additional file 1:** **Table 1.** Consolidated criteria for reporting qualitative studies (COREQ): 32 item checklist.

**Additional file 2.** Interview guide.

## Data Availability

All data generated or analysed during this study are included in this published article.
