# Supplementary material for: Suspension of face-to-face teaching and ad hoc transition to digital learning under Covid-19 conditions – a qualitative study among dental students and lecturers
Source: BMC Med Educ. 2022 Apr 8;22:257. doi: 10.1186/s12909-022-03335-5 (PMC8992419; doi:10.1186/s12909-022-03335-5)
Supplement: Supplementary file 1 — Additional file 1: Table 1. Consolidated criteria for reporting qualitative studies (COREQ): 32 item checklist. [file 12909_2022_3335_MOESM1_ESM.pdf]

Table 1 Consolidated criteria for reporting qualitative studies (COREQ): 32-item checklist

No	Item	Guide questions/description
**Domain 1: Research team and reflexivity**		
Personal Characteristics		
1.	Interviewer/facilitator	Which author/s conducted the interview or focus group? p. 5
2.	Credentials	What were the researcher's credentials? *E.g. PhD, MD* > biographical background
3.	Occupation	What was their occupation at the time of the study? p. 6
4.	Gender	Was the researcher male or female? p. 5
5.	Experience and training	What experience or training did the researcher have? p. 5
Relationship with participants		
6.	Relationship established	Was a relationship established prior to study commencement? no
7.	Participant knowledge of the interviewer	What did the participants know about the researcher? e.g. *personal goals, reasons for doing the research* professional background
8.	Interviewer characteristics	What characteristics were reported about the interviewer/facilitator? e.g. *Bias, assumptions, reasons and interests in the research topic* p. 17
**Domain 2: study design**		
Theoretical framework		
9.	Methodological orientation and Theory	What methodological orientation was stated to underpin the study? *e.g. grounded theory, discourse analysis, ethnography, phenomenology, content analysis* p. 6
Participant selection		
10.	Sampling	How were participants selected? *e.g. purposive, convenience, consecutive, snowball* p. 5
11.	Method of approach	How were participants approached? e.g. *face-to-face, telephone, mail, email* p. 5
12.	Sample size	How many participants were in the study? p. 5 / p. 7
13.	Non-participation	How many people refused to participate or dropped out? Reasons? p. 7
Setting		
14.	Setting of data collection	Where was the data collected? e.g. *home, clinic, workplace* p. 5
15.	Presence of non-participants	Was anyone else present besides the participants and researchers? no
16.	Description of sample	What are the important characteristics of the sample? *e.g. demographic data, date* p. 7
Data collection		
17.	Interview guide	Were questions, prompts, guides provided by the authors? Was it pilot tested? p. 6
18.	Repeat interviews	Were repeat interviews carried out? If yes, how many? no
19.	Audio/visual recording	Did the research use audio or visual recording to collect the data? p. 7
20.	Field notes	Were field notes made during and/or after the interview or focus group? yes
21.	Duration	What was the duration of the interviews or focus group? p. 7
22.	Data saturation	Was data saturation discussed? p. 17
23.	Transcripts returned	Were transcripts returned to participants for comment and/or correction? no
**Domain 3: analysis and findingsz**		
Data analysis		
24.	Number of data coders	How many data coders coded the data? p. 6
25.	Description of the coding tree	Did authors provide a description of the coding tree? p. 7 - 13
26.	Derivation of themes	Were themes identified in advance or derived from the data? p. 7 - 13
27.	Software	What software, if applicable, was used to manage the data? p. 6
28.	Participant checking	Did participants provide feedback on the findings? no
Reporting		
29.	Quotations presented	Were participant quotations presented to illustrate the themes / findings? Was each quotation identified? e.g. *participant number* p. 7 – 13
30.	Data and findings consistent	Was there consistency between the data presented and the findings? yes, p. 7 - 13
31.	Clarity of major themes	Were major themes clearly presented in the findings? p. 7 - 13
32.	Clarity of minor themes	Is there a description of diverse cases or discussion of minor themes? p. 7 - 13
