# Supplementary material for: Suspension of face-to-face teaching and ad hoc transition to digital learning under Covid-19 conditions – a qualitative study among dental students and lecturers
Source: BMC Med Educ. 2022 Apr 8;22:257. doi: 10.1186/s12909-022-03335-5 (PMC8992419; doi:10.1186/s12909-022-03335-5)
Supplement: Supplementary file 2 — Additional file 2. Interview guide. [file 12909_2022_3335_MOESM2_ESM.pdf]

Interview guide

Topic: Experiences of the suspension of face-to-face teaching

- How did you find out about the suspension of face-to-face teaching?
- What immediately went through your head?
- How did you perceive this suspension?
- In your opinion, how did the dental clinic / teaching staff react to the situation?

Topic: Experiences of ad hoc digitalisation

The information then came that the courses should be digitized:

- How did you get this information?
- What did you spontaneously think about studying dentistry?
- How did the technical implementation work for you?
- How do you rate the changed structure of the courses?
- Do you have the impression that you have learned more, less or the same amount?
- How do you rate this?

General: Which moments did you find positive?
